# Impact of the Whole Genome Duplication Event on PYK Activity and Effects of a PYK1 Mutation on Metabolism in *S. cerevisiae*

**DOI:** 10.3389/fmolb.2021.656461

**Published:** 2021-03-16

**Authors:** Hong Chen, Jamie E. Blum, Anna Thalacker-Mercer, Zhenglong Gu

**Affiliations:** ^1^Division of Nutritional Sciences, Cornell University, Ithaca, NY, United States; ^2^Department of Cell, Developmental and Integrative Biology, University of Alabama at Birmingham, Birmingham, AL, United States

**Keywords:** PYK gene, Crabtree effect, whole genome duplication (WGD), yeast, metabolism

## Abstract

**Background:** Evolution of aerobic fermentation (crabtree effect) in yeast is associated with the whole genome duplication (WGD) event, suggesting that duplication of certain genes may have altered yeast metabolism. The pyruvate kinase (PYK) gene is associated with alterations in cell metabolism, and duplicated during the WGD, generating PYK1 and PYK2. Thus, the impact of WGD on PYK activity and role of PYK in yeast metabolism were explored.

**Methods:** PYK activity in the presence or absence of fructose-1,6-bisphosphate (FBP) was compared between pre- and post-WGD yeast. Glucose consumption, ethanol production, and oxygen consumption were measured in wildtype yeast and yeast with a T403E point mutation, which alters FBP binding affinity.

**Results:** FBP stimulated increased PYK activity in pre-WGD yeast and in the PYK1 isoforms of post-WGD yeast, but not in the PYK2 isoforms of post-WGD yeast. Compared to wildtype, T403E mutant yeast displayed reduced glucose consumption, reduced ethanol production, and increased mitochondrial metabolism.

**Conclusion:** The WGD event impacted the sensitivity of PYK activity to FBP. Mutations in the FBP binding domain of PYK induce metabolic shifts that favor respiration and suppress fermentation.

## Introduction

Organismal survival depends on the balance of substrate flux through energy-generating pathways and the adaptation of these metabolic pathways to environmental changes. Glucose is a common energy substrate that can be fermented into lactate/ethanol or metabolized aerobically through mitochondrial respiration. While anoxic conditions require fermentation to sustain ATP production, fermentation can also occur in aerobic conditions, a phenomenon called the Warburg effect (mammalian cells) or Crabtree effect (yeast). While aerobic fermentation yields less ATP per molecule of glucose than mitochondrial respiration, its advantages include a faster rate of ATP production and sustained NAD availability to support glucose flux into biosynthetic pathways (Pfeiffer and Morley, 2014; Liberti and Locasale, 2016).

Evolution of the Crabtree effect in yeast is associated with the whole genome duplication event (WGD) (Merico et al., 2007), in which many genes duplicated, conferring dosage effects of increased copy number and opportunities for previously shared functions to become split between two genes (subfunctionalization) or new functions to emerge (neofunctionalization) (Chen et al., 2008). Despite the association between WGD and the Crabtree effect, evidence linking duplication of specific genes with development of the Crabtree effect is sparse. Several observations suggest a relationship between the Crabtree effect and duplication of the pyruvate kinase (PYK) gene. PYK encodes the enzyme that catalyzes the final step in glycolysis, the conversion of phosphoenolpyruvate (PEP) into pyruvate. WGD yielded two isoforms of PYK: PYK1 and PYK2. PYK1 is allosterically regulated in response to metabolites such as fructose-1,6-bisphosphate (FBP), and PYK2 is a constitutively active isoform (Boles et al., 1997). Yeast with low PYK activity (through selective expression of PYK1 or PYK2) grow at a slower rate than yeast with high PYK activity and have increased oxygen uptake (Gruning et al., 2011). Additionally, a creative effort to block ethanol production and sustain cell growth using only glucose as a carbon source in yeast that were engineered to produce lipids, through deleting pyruvate decarboxylase (PDC), an enzyme involved in fermentation of pyruvate to ethanol, required simultaneous loss-of-function mutations in PYK1 (Yu et al., 2018). It is likely that loss-of-function mutations in PYK1 allowed for increased mitochondrial respiration necessary for growth under these conditions. Taken together, previous research suggests that PYK1 balances glycolytic flux into fermentation and respiration, key aspects of the Crabtree effect.

In this report, we compared PYK activity between pre-WGD and post-WGD yeast species to determine how activity and FBP regulation were impacted during the WGD and evolution of the Crabtree effect. Additionally, to determine whether the FBP binding capacity of PYK1 is necessary for its role in the Crabtree effect, we compared metabolic parameters between wildtype and PYK1 mutant yeast that lack FBP binding capability.

## Methods

### Cloning and Purification of Pyruvate Kinase Genes

We independently cloned eight PYK genes onto ppSUMO Kan plasmids. Sequences were obtained from the Saccharomyces genome database. These genes and the corresponding cloning primers are: *Saccharomyces cerevisiae* PYK1 (5′CAGATTGGTGGATCCATGTCTAGATTAGAAAGATT3′ and 5′GTGGTGGTGCTCGAGTTAAACGGTAGAGACTTG CA3′)/PYK2 (5′CAGATTGGTGGATCCATGCCAGAGTCCAG ATTGCA3′ and 5′GTGGTGGTGCTCGAGCTAGAATTCTTGA CCAACA3′), *Saccharomyces baynus* PYK1 (5′CAGATTG GTGGATCCATGTCTAGATTAGAAAGAT3′ and 5′GTGG TGGTGCTCGAGTTAAACAGTAGAGACTTGCA3′)/PYK2 (5′ CAGATTGGTGGATCCATGCCAGAATCAAGACTGC3′ and 5′GTGGTGGTGCTCGAGCTAAAACTCTTCACCAA3′), *Kluy- veromyces polysporus* PYK (5′CAGATTGGTGGATCC ATGATGCAATCTAGATTAGG3′ and 5′GTGGTGGTG CTCGAGTTAAACAATGGTAACTCTCA3′), *Kluyveromyces lactis* PYK (5′CAGATTGGTGGATCCATGGAATCTAGATTAG GCTG 3′ and 5′GTGGTGGTGCTCGAGTTAAACGGTCAAGA CACGTA 3′), *Saccharomyces kluyveri* PYK (5′CAGATTGGT GGATCCATGGAATCTAGACTAGGTTG3′ and 5′GT GGTGGTGCTCGAGTTAGACGGTCAAGACACGCA3′), and *Candida albicans* PYK (5′CAGATTGGTGGATCCATGTCTCAC TCATCTTTATC3′ and 5′GTGGTGGTGCTCGAGTTAAGCT TGGACGATTCTAA3′). Each PYK sequence was cloned as a *Bam*HI-*Xho*I fragment (sites are underlined in the sequence of the primers) into the same plasmid.

PYK protein was purified from *Escherichia coli* DH5a containing each ppSUMO-PYK vector. Overnight cultures were transferred to fresh Lennox Broth + kanamycin media and grown up to ∼0.6 OD at 37°C. Then the temperature was lowered to 25°C, cells were incubated for an additional 0.5 h, 1 M isopropyl beta-D-1-thiogalactopyranoside (IPTG) was added to stimulate protein expression and cells were incubated for 4 h at 25°C. Cells were then collected and stored at −80°C.

Cells were sonicated in cold PBS and mixed with washed His-sumo tagged beads (GOLDBIO, CAT #H-310-5) at 4°C for 1 h. The beads were then washed with PBS buffer several times and protein was quantified using a Bradford assay (Bio-Rad, CAT #500-0006). Washing continued until no protein was detected in the washed elution. Then the beads were transferred to a 2 ml tube with additional fresh PBS and the restriction enzyme ulpl, and incubated with mild shaking at 4°C overnight. The resulting suspension containing purified PYK was stored at −80°C until the assay was performed.

### Pyruvate Kinase Assay

Pyruvate kinase activity was measured by a continuous assay coupled to lactate dehydrogenase (LDH) as previously described (Mesecar and Nowak, 1997). Activity was measured as the change in absorbance at 340 due to oxidation of NADH by LDH. Spectrophotometric measurements were made using a Cary 100 UV/VIS spectrophotometer with a cuvette holder thermocoupled at room temperature. The assay buffer was 0.05 M Imidazole-HCl buffer pH 7.6, 0.12 M potassium chloride, 0.062 M magnesium sulfate, 4.5 mM adenosine diphosphate, 6.6 mM NADH, 1 mM PEP, and ∼10 unit/ml LDH; 1 mM FBP was added when indicated. The reaction volume was 3 mL. Reactions were initiated by the addition of PYK. The slope of change in absorbance over time was measured from 3 to 7 min. This change in absorbance was normalized to total PYK concentration, which was assessed with a Bradford assay (Bio-Rad). Three replicates were measured.

### Generation of T403E Mutant Strain

A vector carrying PYK1 with a T403E mutation was kindly provided by B. L. Stoddard (Bond et al., 2000). A PCR fragment was amplified using this plasmid as the template and two primers: 5′CTTGTTTCTATTTACAAGACACCAATCAAAACAAATAA AACATCATCACAATGTCTAGATTAGAAAGATT3′ and 5′TG GCTAGAAGAATAGGACGGAGTAGCACTTATCGTTTCTGA AAGAGGATCAATCCTTTGAAATCTTGAA3′. This fragment, which contains the latter part of *S. cerevisiae* PYK1 with the T403E mutation and a following kanMX marker, was then transferred into a PYK2 deleted BY4742 strain (MATa his3Δ leu2Δ lys2Δ ura3Δ pyk2Δ). The transformed clones were picked and sequenced for the PYK1 gene. Clones with the PYK1 T403E mutation were kept, and clones with wildtype PYK1 were kept and used as controls.

### Glucose and Ethanol Measurements

Three independent clones for each strain were picked. The overnight cultures were transferred into 2% YPD media and grown for 2 days. Collected cells were washed with distilled water and then grown in 10 ml SDC medium containing 20% glucose in a 150 ml flask at 250 r/min and 30°C. In these experiments, a high cell concentration was used (OD_600_ reading over 15) to rapidly evaluate fermentation and minimize effects of cell growth. Sample media was collected every 4 h for HPLC analysis. The OD_600_ was monitored during the procedure to ensure cell density did not change.

A Waters Associates liquid chromatograph (model ALC/GPC 244) equipped with a RID detector and an H-column (3.2 mm i.d. × 20 cm) was used to measure the concentration of glucose and ethanol. The mobile phase was 5% H_2_SO_4_. Operating conditions were flowrate: 0.6 ml/min, detector sensitivity: 8X, and chart speed: 0.4 in/min. Five standards containing both glucose and ethanol were run with glucose concentrations ranging from 100, 50, 10, and 1 to 0.5 g/L, and ethanol concentrations ranging from 50, 20, 10, and 1 to 0.5 g/L. Glucose consumed was calculated by subtracting the measured glucose concentration from the starting concentration (200 g/L) and used to calculate the ratio of ethanol produced: glucose consumed.

### Oxygen Consumption Measurement

Yeast were grown to an OD_600_ of 1 in 2% YPD, centrifuged, and kept on ice. Before the measurements, yeast were resuspended in SD–2% glucose and put on a shaker at 30°C for 1 h. OD_600_ measurements and cell counts were performed immediately before putting yeast in the oxygen sensing setup. The oxygen depletion rate was monitored by using a lab-built respirometer comprising a polarographic dissolved oxygen probe of Orion Star^TM^ Series DO Meter (Thermo Scientific) in a glass syringe with constant agitation (Lamboursain et al., 2002). Oxygen consumption rates were expressed as mg/ml O_2_ per minute per 1 × 10^7^ cells. Measurements were performed on three replicates of mixed clones.

### Statistics

All statistical analyses were performed in GraphPad Prism 8. PYK activity was analyzed using a two-way ANOVA with main effects of yeast strain and FBP and a yeast strain by FBP effect. Sidak’s multiple comparisons test was used for *post hoc* analysis to assess the impact of FBP within each yeast strain. Glucose levels in the media, ethanol production, and the ethanol production: glucose consumption ratio were compared between wildtype and T403E mutant yeast using a two-way repeated measures ANOVA test, with main effects of time and yeast strain, and a time by strain interaction effect. Sidak’s multiple comparisons test was used for *post hoc* analysis to assess the impact of yeast strain within each time. Oxygen consumption rate was compared between wildtype and T403E mutant yeast using an unpaired *t*-test.

## Results

**FIGURE 1:**
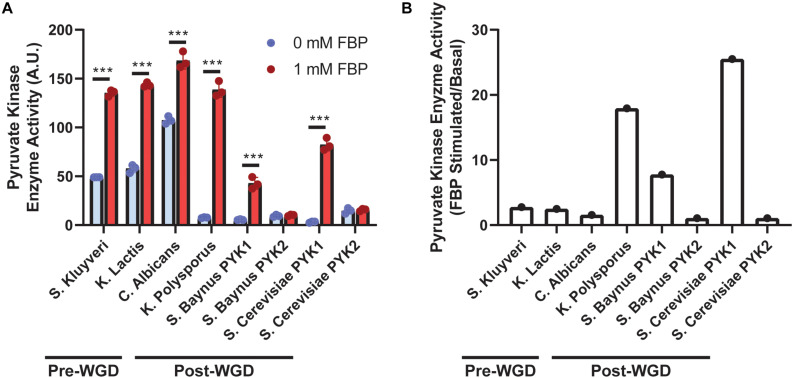
Pyruvate kinase (PYK) activity across yeast species. Activity of PYK isoforms **(A)** isolated from different yeast species was measured in the presence or absence of FBP (*n* = 3/group). Ratio of FBP stimulated to basal PYK activity **(B)** (*n* = 1). WGD denotes whole genome duplication. A.U. denotes arbitrary units. Blue bars indicate 0 mM FBP, and red bars indicate 1 mM FBP included in the reaction. *** denotes *p* < 0.001.

To assess the impact of WGD and PYK duplication on PYK activity, basal and FBP stimulated PYK activity was compared between pre- and post-WGD yeast species (Figure 1A). *S. kluyveri*, *C. albicans*, and *K. lactis* were selected as representative pre-WGD yeast species, and *K. polysporus*, *S. baynus*, and *S. cerevisiae* were selected as representative post-WGD yeast species. Interestingly, *K. polysporus* retained only one PYK gene after the WGD event, it is unclear whether the other PYK gene was lost during subsequent evolution. *C. albicans* and *K. lactis* are crabtree negative yeast (Gonzalez-Siso et al., 2000; Moller et al., 2001; Broxton et al., 2018). *S. kluyveri* is intermediate in aerobic fermentation (Moller et al., 2001). *S. baynus*, *K. polysporus*, and *S. cerevisiae* are crabtree positive yeast (Merico et al., 2007; Christen and Sauer, 2011; Hagman et al., 2014).

PYK activity was increased by FBP in yeast species with only one PYK copy [*S. kluyveri*, *K. lactis*, *C. albicans*, and *K. polysporus* (*p* < 0.001)]. Further, as anticipated, FBP increased activity of PYK1 isoforms (*p* < 0.001), but not PYK2 isoforms (*p* > 0.99). Additionally, the impact of FBP on PYK activity was more robust in PYK1 isoforms of post-WGD yeast (7.8-fold for *S. baynus* and 25.5-fold for *S. cerevisiae*) or *K. polysporus*, the post-WGD yeast with one PYK copy (17.9-fold) compared to pre-WGD yeast (2.8-fold *S. kluyveri*, 2.5-fold *K. lactis*, and 1.6-fold *C. albicans*) (Figure 1B).

**FIGURE 2:**
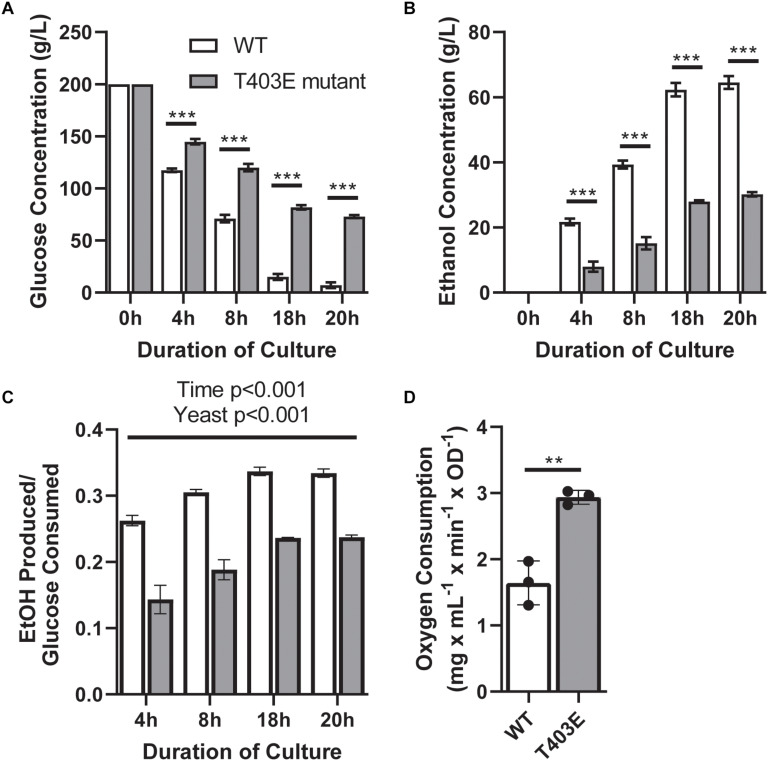
Metabolic parameters in wildtype (WT) and T403E mutant yeast. Media glucose **(A)**, media ethanol **(B)**, ethanol:glucose ratio **(C)**, and oxygen consumption **(D)** were measured in WT and T403E mutant yeast (*n* = 3/group each). White bars indicate WT, and gray bars indicate T403E mutant. ** denotes *p* < 0.01 and *** denotes *p* < 0.001.

To address the contribution of the FBP binding capacity of PYK1 to overall metabolic phenotype, a T403E point mutation was generated in the PYK1 gene in *S. cerevisiae* on a PYK2 deletion background. The T403E mutation prevents FBP binding and thus prevents allosteric activation of PYK1 (Bond et al., 2000; Fenton and Blair, 2002). Compared to wildtype, the T403E mutant yeast showed greater glucose concentrations in the media at each time point, indicating lower glucose consumption (Figure 2A, *p* < 0.0001). The T403E mutant also demonstrated reduced ethanol secretion (Figure 2B, *p* < 0.0001). Importantly, the T403E mutant yeast had a lower ethanol:glucose ratio (Figure 2C, *p* < 0.05) consistent with lower aerobic fermentation. Additionally, and in line with a metabolic shift, the T403E mutant yeast had a higher oxygen consumption rate compared to wildtype (Figure 2D, *p* = 0.003), suggesting increased rates of mitochondrial respiration.

## Discussion

Excitingly, this research furthers understanding of the relationship between the WGD and evolution of PYK. FBP increased activity of PYK isoforms from yeast with only one PYK gene and PYK1, but not PYK2. This confirms that PYK2 is a constitutively active isoform. Further, the PYK1 isoforms of post-WGD yeast, and PYK in *K. polysporus*, are more sensitive to FBP compared to the pre-WGD yeast PYK isoforms. Thus, the WGD may have increased the sensitivity of PYK (PYK1 in yeast with two isoforms) to FBP, in addition to creating an insensitive isoform.

This research identified that mutations in the FBP binding domain of PYK1 shift *S. cerevisiae* metabolism from fermentation to respiration. This point mutation may influence fermentation capacity either through specifically regulating FBP binding capacity or through decreasing overall PYK activity as a consequence of blocking FBP activated activity. We hypothesize that the first explanation is more likely, since we observed that pre-WGD yeast have higher PYK activity compared to post-WGD, Crabtree positive yeast. Of note, yeast with the PYK1 mutation still possessed the capacity for aerobic fermentation, highlighting that mutating the FBP binding domain of PYK1 does not abolish the Crabtree effect. Indeed, there is evidence that the Crabtree effect evolved in multiple stages, with the origin of aerobic fermentation in yeast predating the WGD, though the amount of ethanol produced under aerobic conditions increased sharply concurrent with the WGD (Hagman et al., 2013).

The observed relationship between PYK1 and metabolism aligns with a recent genome-wide association study (GWAS) that found PYK1 as a key regulator of *Schizosaccharomyces pombe* yeast growth when oxidative phosphorylation is blocked (Kamrad et al., 2020). Specifically, T343A mutant yeast, which have greater PYK1 activity, are more resistant to the growth inhibiting effects of antimycin A, a mitochondrial complex III inhibitor (Kamrad et al., 2020). This suggests that “A” mutant yeast rely less on respiration. Indeed, the researchers found that yeast strains with the “A” mutation compared to the wildtype “T” show greater glucose uptake and lower oxygen consumption (Kamrad et al., 2020). Thus, pairing our result with recent research shows that either increasing or decreasing PYK1 activity alters fermentation, cementing the role of PYK1 in promoting fermentation in yeast. Synthesis and assembly of the systems controlling mitochondrial respiration and oxidative phosphorylation require genes located in both the nuclear and the mitochondrial genome (Surguchov, 1987). Further studies are needed to better understand signaling pathways that mediate this nuclear-mitochondrial crosstalk.

Similar to yeast, in mammalian cells, pyruvate kinase is encoded by multiple isoforms, which intriguingly show tissue-specific expression patterns. The isoforms are PKL in the liver and kidney, PKR in red blood cells, PKM1 in many terminally differentiated tissues such as brain and skeletal muscle, and PKM2 in many dividing cell populations such as stem cells and tumor cells. PKM1 and PKM2 show similar properties as PYK2 and PYK1, respectively. For example, both PYK1 and PKM2 are sensitive to activation by FBP, form a tetrameric complex for PEP to pyruvate conversion (Anastasiou et al., 2012; Li et al., 2015), are implicated in regulation of serine synthesis (Ye et al., 2012; Li et al., 2015) and oxidative stress (Anastasiou et al., 2011; Yu et al., 2017; Kamrad et al., 2020), and are partly localized to the nucleus (Yang and Lu, 2013; Li et al., 2015).

Of note, the relationship between PKM2 and fermentation/respiration is heterogeneous between cell types. For example, in lung cancer cells, PKM2 knockdown reduces lactate secretion (Prakasam et al., 2017). Interestingly, mitochondrial mass and membrane potential were increased in one lung cancer line (H1299), but not another (A549) (Prakasam et al., 2017). Oppositely, in hepatocellular carcinoma cells with a poly(adp-ribose) polymerase family member 14 (PARP14) knockdown, knockdown of PKM2 increased lactate production (Iansante et al., 2015). Whether similar variation exists between yeast strains or can be introduced by environmental variation is unknown. Additionally, PKM2 interacts with a range of protein binding partners to promote phenotypes beyond metabolism such as cell proliferation (Lunt et al., 2015) and inflammation (Alves-Filho and Palsson-Mcdermott, 2016).

Future research into the interactome of PYK1 will further understanding of coordination of yeast fermentation with other cellular processes. In summary, this research identified alterations in the sensitivity of PYK toward FBP as a result of the WGD and showed that PYK1 promotes aerobic fermentation, a key aspect of the Crabtree effect, in *S. cerevisiae* yeast.

## Data Availability Statement

The original contributions presented in the study are included in the article/Supplementary Material. Further inquiries can be directed to the corresponding author/s.

## Author Contributions

HC and ZG: conceptualization. HC: data collection. JB: data analysis and figure preparation. HC, JB, AT-M, and ZG: data interpretation and manuscript preparation. ZG and AT-M: supervision. All authors contributed to the article and approved the submitted version.

## Conflict of Interest

The authors declare that the research was conducted in the absence of any commercial or financial relationships that could be construed as a potential conflict of interest.

## Supplementary Material

Supplementary Table 1 Manuscript data.
